# Identification and characterization of serovar-independent immunogens in *Actinobacillus pleuropneumoniae*

**DOI:** 10.1186/s13567-017-0479-5

**Published:** 2017-11-09

**Authors:** Fabio Antenucci, Cyrielle Fougeroux, Janine T. Bossé, Zofia Magnowska, Camille Roesch, Paul Langford, Peter Johannes Holst, Anders Miki Bojesen

**Affiliations:** 1 Department of Veterinary and Animal Sciences, University of Copenhagen, Stigbøjlen 4, 1870 Frb. C., 1-20, Building: 301, Copenhagen, Denmark; 2 Department of International Health, Immunology and Microbiology ISIM, University of Copenhagen, Øster Farigmagsgade 5, Bldg 22/23, København K, 1014 Copenhagen, Denmark; 3 Department of Medicine, St Mary’s Campus, Imperial College London, 236 Wright Fleming Wing, London, UK; 4 Izon Science Ltd, Bâtiment Laennec, 60 Avenue Rockefeller, 69008 Lyon, France

## Abstract

Despite numerous actions to prevent disease, *Actinobacillus pleuropneumoniae* (*A. pleuropneumoniae*) remains a major cause of porcine pleuropneumonia, resulting in economic losses to the swine industry worldwide. In this paper, we describe the utilization of a reverse vaccinology approach for the selection and in vitro testing of serovar-independent *A. pleuropneumoniae* immunogens. Potential immunogens were identified in the complete genomes of three *A. pleuropneumoniae* strains belonging to different serovars using the following parameters: predicted outer-membrane subcellular localization; ≤ 1 trans-membrane helices; presence of a signal peptide in the protein sequence; presence in all known *A. pleuropneumoniae* genomes; homology with other well characterized factors with relevant data regarding immunogenicity/protective potential. Using this approach, we selected the proteins ApfA and VacJ to be expressed and further characterized, both in silico and in vitro. Additionally, we analysed outer membrane vesicles (OMVs) of *A. pleuropneumoniae* MIDG2331 as potential immunogens, and compared deletions in *degS* and *nlpI* for increasing yields of OMVs compared to the parental strain. Our results indicated that ApfA and VacJ are highly conserved proteins, naturally expressed during infection by all *A. pleuropneumoniae* serovars tested. Furthermore, OMVs, ApfA and VacJ were shown to possess a high immunogenic potential in vitro. These findings favour the immunogen selection protocol used, and suggest that OMVs, along with ApfA and VacJ, could represent effective immunogens for the prevention of *A. pleuropneumoniae* infections in a serovar-independent manner. This hypothesis is nonetheless predictive in nature, and in vivo testing in a relevant animal model will be necessary to verify its validity.

**Electronic supplementary material:**

The online version of this article (10.1186/s13567-017-0479-5) contains supplementary material, which is available to authorized users.

## Introduction

*Actinobacillus* *pleuropneumoniae* (*A. pleuropneumoniae*) is a Gram-negative bacterium causing respiratory disease in pigs worldwide. *A. pleuropneumoniae* outbreaks are a constant threat to the pig farming industry, and are estimated to reduce the expected revenues in pig production by an average of 6.4€ per pig in affected herds [1]. To date, *A. pleuropneumoniae* has been divided into 16 serovars and 2 biovars, according to capsular antigens and nicotinamide dinucleotide (NAD) requirements [2–4], respectively. The spatiotemporal prevalence of individual serovars has been shown to vary, both regionally and over time [5].

The first generation of vaccines developed against *A. pleuropneumoniae* was based on inactivated whole cells (bacterins). Although in some cases effective in preventing both colonization and morbidity, these vaccines offered protection against little more than the specific serovar used for immunization [6, 7]. A step forward was made with the characterization of the Apx toxins, a set of four pore-forming cytolysins (Apx I-IV) centrally involved in *A. pleuropneumoniae* pathogenesis, and secreted by all *A. pleuropneumoniae* serovars in various combinations [3, 8]. Vaccines based on Apx toxins elicit a strong humoral response and are able to effectively reduce morbidity in vaccinated animals [9–12], but fail in preventing colonization and thus create the risk of the spreading of disease by asymptomatic carriers [6, 11, 13]. Nonetheless, vaccines based on Apx toxins in combination with various outer membrane (OM) proteins (see [6] for a complete review of *A. pleuropneumoniae* OM immunogens) are the most widely used for the prevention of *A. pleuropneumoniae* infection. Thus, despite the availability of vaccines for *A. pleuropneumoniae*, their efficacy is challenged by limited prevention of host colonization [10, 14] or cross-serovar protection [11, 13, 15]. Hence a major challenge impeding development of an effective vaccine has been to identify immunogens capable of preventing colonization of the host by all known *A. pleuropneumoniae* serovars as well as the severity of the disease.

The selection of immunogens for vaccine development has for decades been a rather laborious process, usually requiring the isolation and testing of hundreds of potential immunogens from cultures with subsequent identification of the genes involved in their expression, and finally production of the chosen immunogens for in vitro and in vivo testing [16]. The advent of bioinformatics and the sequencing of bacterial genomes have allowed researchers to invert this workflow and identify instead potential immunogens at a genetic level. Once identified, candidate immunogens can be easily expressed using suitable vectors for subsequent testing. This alternative workflow has quite fittingly been called reverse vaccinology (RV) [17, 18], and has offered immunologists the opportunity to significantly reduce the workload traditionally necessary for vaccine development [19].

Similar to RV, bacterial outer membrane vesicles (OMVs) represent a revolution in the way non-live vaccines are now conceived and developed. The OMVs are vesicles secreted by the majority of Gram-negative bacteria that serve numerous biological functions, from cargo delivery to immune modulation [20, 21]. The OMVs are secreted during all phases of bacterial growth, and vary in size between 20 and 300 nm for Gram-negative bacteria [22, 23]. Secretion of OMVs has been proven to be negatively regulated by various genes, inactivation of which often leads to an enhanced secretion of OMVs [24]. Being released from the bacterial OM, OMVs generally represent antigenic properties very similar to intact bacteria. In addition, OMVs have a significant adjuvant potential due to the presence of lipopolysaccharides (LPS) and immunomodulatory proteins on their surface [25, 26]. The relatively high molecular mass of OMVs also allows for more cost-effective purification methods, as compared to recombinant antigens [27], leading to lower vaccine production costs [28, 29]. Additionally, the structure and biogenesis of OMVs offer the possibility to engineer them in order to add desired antigens, either to their surface or as cargo [30, 31]. However, immunization with OMVs also presents some risks. The presence of LPS on OMVs, for example, could pose a threat to the vaccinated individual, potentially causing Systemic Inflammatory Response Syndrome (SIRS) after immunization [32].

Both OMVs and proteins identified using RV are considered promising vaccines commodities, and have so far been utilized commercially in the widely used vaccine against *Neisseria meningitidis* [33]. Here we employ a similar methodology for the identification of serovar-independent immunogens as a first step in the development of a broadly protective vaccine against *A. pleuropneumoniae*.

## Materials and methods

### Bacterial strains, genomes, and sequence analysis

The *A. pleuropneumoniae* strains and accession numbers for genomes used in this investigation are listed in Table 1. Unless otherwise stated, all sequence analysis programmes were used with default parameters. The pan-proteomes extracted from genomes of *A. pleuropneumoniae* JL03, L20 and AP76 strains (serovars 3, 5b, and 7, respectively) were analysed by PSORTb v3.0 [34]. Proteins whose subcellular localization was predicted as “OuterMembrane” with a score > 9.00/10.00 were included in a preliminary candidate immunogen list (Additional file 1). The conservation of candidate immunogen genes across all published *A. pleuropneumoniae* genomes (those available in Genbank as of 14/02/17) was verified using BLASTn. Minimum and maximum conservation rates were noted in the preliminary candidate immunogen list (Additional file 1), as was the predicted presence or lack of a signal peptide, as detected by SignalP 4.1 [35], and the number of transmembrane helices (TMHs), as predicted by the TMHMM Server v. 2.0 [36]. Candidates with > 1 predicted TMH were discarded from further analysis. Preliminary candidate immunogens were screened for available data regarding their biological function and/or immunogenicity, narrowing down the initial list to 9 candidate immunogens (Table 2). Finally, ApfA and VacJ were chosen for expression and characterisation. The ApfA and VacJ three-dimensional protein structures were predicted using the I-TASSER server [37]. Highest confidence models were chosen as predicted structures (ApfA model C-score: −0.40; VacJ model C-score: −3.74).

**Table 1:** ***A. pleuropneumoniae*** **genomes and strains included in this study**

Strain	Serovar	Genome assembly Accession Number (NCBI)	Function
*A. pleuropneumoniae* JL03	3	CP000687.1	In silico identification of potential immunogens
*A. pleuropneumoniae* L20	5b	CP000569.1	
*A. pleuropneumoniae* AP76	7	CP001091.1	
*A. pleuropneumoniae* MIDG2331	8	NZ_LN908249.1	OMV isolation and characterization
*A. pleuropneumoniae* MIDG2331 Δ*nlpI*	8	NA	
*A. pleuropneumoniae* HK361	2	NA	In vitro immunogenicity assessment

NCBI accession numbers are provided.

NA: non available.

**Table 2:** **Candidate immunogen list**

Protein	Accession Number (NCBI)	Function/putative role in virulence
ApfA	YP_001053581.1	Fimbrial subunit protein, demonstrated role in *A. pleuropneumoniae* colonization and virulence [56]
APP7_2042	WP_005616333.1	Putative ligand-gated iron transporter
HecB	ACE61667.1	Hemolysin activation protein and putative virulence factor in *Arcobacter butzleri* [67]
HgbA	YP_001053746.1	Hemoglobin binding protein, *A. pleuropneumoniae* Δ*hgbA* mutants exhibit an attenuated virulence [54]
Irp	ABN74015.1	Iron-regulated OM receptor/transporter possibly involved in hemin transport, upregulated in *A. pleuropneumoniae* after colonisation [58]
OstA	YP_001053661.1	Organic solvent tolerance protein in *Helicobacter pilori*. OstA inactivation causes altered membrane permeability, sensitivity to organic solvent and susceptibility to antibiotics [68]
PepN	ABN74424.1	Multi-subunit transmembrane metalloprotease able to degrade porcine IgA and IgG, expressed during *A. pleuropneumoniae* infection in vivo [55]
SlyB	ABN73145.1	Outer membrane lipoprotein, contributes to cell envelope integrity in *Burkholderia multivorans* [69] and is linked to the stress response to Mg2 + depletion [70]
VacJ	YP_001054603.1	OM lipoprotein, *Pasteurella multocida* VacJ protein moderately immunogenic and protective when inoculated in vivo [63]

NCBI accession numbers are provided.

### Construction of Δ*degS* & Δ*nlpI* mutants

The *A. pleuropneumoniae* serotype 8 MIDG2331 strain was engineered for the construction of individual Δ*nlpI* and Δ*degS* mutants. The *nlpI* or *degS* genes of the wild type (wt) strain were each replaced with a synthesised (Eurofins Genomics) trimethoprim resistance cassette consisting of the *drfA14* gene from plasmid pM3389T [38] under control of the *A. pleuropneumoniae sodC* promoter [39], and followed by the 9 bp sequence required for efficient uptake of DNA during natural transformation of *A. pleuropneumoniae* [40]. Gene replacement constructs were designed by In-Fusion cloning, with the *dfrA14* cassette flanked by approx. 500 bases of sequence upstream and downstream of the gene to be replaced, as previously described [41]. Sequences were amplified using AccuPrime Taq DNA high-fidelity Polymerase (ThermoFisher Scientific). Briefly, primers (*degS*_left_for + *degS*_right_rev; *nlpI*_left_for + *nlpI*_right_rev; Table 3) were designed to amplify the left and right sequences flanking the gene to be deleted, with 15 bp extensions to allow directional cloning into the linear vector pJET1.2/blunt (ThermoFisher Scientific). The resulting plasmids, pJET-*degS* and pJET-*nlpI* were opened by inverse polymerase chain reaction (PCR), using *degS*_left_rev + *degS*_right_for and *nlpI*_left_rev + *nlpI*_right_for primer pairs (Table 3). The *dfrA14* cassette was amplified and cloned into the inverse-amplified pJET-*degS* and pJET-*nlpI* plasmids, using primers with 15 bp extensions matching the ends of the linearized constructs (*dfrA14*_*degS*_for + *dfrA14*_*degS*_rev; *dfrA14*_*nlpI*_for + *dfrA14*_*nlpI*_rev; Table 3). The resulting plasmids, pJETΔ*degS*::*dfrA14* and pJETΔ*nlpI*::*dfrA14* were linearized by digestion with FastDigest XbaI restriction enzyme (ThermoFisher Scientific) prior to natural transformation into *A. pleuropneumoniae* serotype 8 strain MIDG2331 [42], as previously described [41]. Transformants were selected on BHI (brain–heart infusion)-NAD containing 10 μg/mL trimethoprim, and the gene replacements were confirmed by PCR using primers designed to verify the location of the *dfrA14* cassette insertion (*degS*_test + *dfrA14*_test_for; *nlpI*_test + *dfrA14*_test_rev; see Table 3).

**Table 3:** **Primers used in this study**

Primer	Sequence (5′– > 3′)
*degS*_left_for	GAATTCCTGCAGCCCGCGAACGGCTAAATCTATATGATG
*degS*_left_rev	CCAAGGTTGAAACGAAACCTAGTGCAATCGCTTGTAC
*degS*_right_for	TTGACGGAGGGCTTTGGCGAATTTCCGGAACTATAATGC
*degS*_right_rev	ACTAGTGGATCCCCCCGCAACCTCACGATTTCTATCTC
*nlpI*_left-for	GAATTCCTGCAGCCCGCGTGATGGAACAAGCGATTC
*nlpI*_left-rev	CCAAGGTTGAAACGAGCATCAAGAAGCGAAGGTGAAAC
*nlpI*_right-for	TTGACGGAGGGCTTTACGTGGTGGTAGCAATGTTG
*nlpI*_right-rev	ACTAGTGGATCCCCCTTGTAGTGCAGAGAGGTCTAACG
*dfrA14*-*degS*_for	CGATTGCACTAGGTTTCGTTTCAACCTTGGTGTTTGG
*dfrA14*-*degS*_rev	TTCCGGAAATTCGCCAAAGCCCTCCGTCAAATTTATTACC
*dfrA14*-*nlpI*_for	TTCGCTTCTTGATGCTCGTTTCAACCTTGGTGTTTGG
*dfrA14*-*nlpI*_rev	TTGCTACCACCACGTAAAGCCCTCCGTCAAATTTATTACC
*degS*_test	AATAATGACCGAACACATCC
*nlpI*_test	ATTTCGTCCGCTTCATCC
*dfrA14*_test-for	TCGTTTCAACCTTGGTGTTTGG
*dfrA14*_test-rev	AAAGCCCTCCGTCAATTTTATTACC
*apfA*_pET44_for	GTCCCaCGaGGaAGCCAgAAgCTAAGTCTTATTCGACCa
*apfA*_pET44_rev	ACTTcATTAACATTAGTTTATCGCgCAGAAATTTGCC
pET44_*apfA*_rev	AAGACTTAGcTTcTGGCTtCCtCGtGGGACCAG
pET44_*apfA*_for	TTCTGcGCGATAAAcTAATGTTAATgAAGTTGGGCGTTCCT
*vacJ*_pET44_for	GTCCCaCGaGGaAGCAAgTTAAAgCAATTAAGgTTAGTAGCC
*vacJ*_pET44_rev	ACTTcATTAACATTAATCAATgTCTTTcAATTCTTCTTCGG
pET44_*vacJ*_rev	TAATTGcTTTAAcTTGCTtCCtCGtGGGACCAG
pET44_*vacJ*_for	TTgAAAGAcATTGATTAATGTTAATgAAGTTGGGCGTTCCT

Lower case letters indicate nucleotides modified from the target sequence in order to reduce primer secondary structure formation.

### OMV isolation and analysis

For isolation of OMVs from *A. pleuropneumoniae* MIDG2331 wt, *ΔdegS* and Δ*nlpI* clones, 600 mL of BHI-NAD broth was inoculated for each with a 1/10 volume of overnight (ON) culture (to an initial OD_600_ = 0.1 (OD: optical density) and incubated at 37 °C/200 rpm for approx. 7 h (late exponential phase). Culture supernatants, obtained following centrifugation (6000 *g*, 20 min, 4 °C), were filtered through 0.45 μm Minisart sterile filters (Sartorius) before concentration of OMVs by Hydrostatic Filtration (HF), using a modified version of the protocol described by Musante et al. [43]. Briefly, for each sample, the filtered supernatant was loaded into a dialysis membrane (Biotech CE Dialysis Tubing, 1000 kD molecular weight cut-off (MWCO), 31 mm Flat-width; Spectrum Labs) and encased vertically inside a Plexiglas column, where the hydrostatic pressure of the fluid itself provided the force needed to concentrate all particles above the membrane’s MWCO. The column, sealed with transparent film at the top to help prevent dehydration of the membrane, and with a bottle below to collect the flow through, was placed at 4 °C ON. The concentrated supernatant was recovered from inside the membrane the following day, filtered through a 0.45 μm filter, and re-suspended in sterile phosphate buffered saline (PBS) to a final volume of 300 mL prior to being re-concentrated in a second round of HF. The following day, the concentrate was filtered through a 0.45 μm filter and statically dialysed 1:1000 in sterile PBS (3 h, 4 °C). The dialysed samples were then further concentrated using Amicon Ultra-15 Centrifugal Filter Units (Merck Millipore) to a final volume of 3 mL (i.e. 200-fold concentration).

Samples were analysed by transmission electron microscopy (TEM). Carbon-coated laminae were suspended into the OMV solutions for a few seconds and soaked with a droplet of 2% (w/v) phosphotungstic acid. Grids were soaked into a Sellotape/Chlorophorm solution to increase adherence and then placed on the laminae. After 1–2 min, the Grids were removed and left to dry on filter paper. Once dried, stained grids were examined using a Philips CM100 Transmission Electron Microscope (Philips). Additionally, aliquots of the OMV samples were quantified by tunable resistive pulse sensing (TRPS) using a qNano device (Izon Sciences Ltd) following the protocol recommended by the operator [44]. Data were analyzed using the proprietary data capture and analysis software, Izon Control Suite V.3.3.2.2001.

### Expression and purification of recombinant ApfA and VacJ

The *apfA* and *vacJ* genes were amplified from genomic DNA using Phusion Hot Start II DNA high-fidelity Polymerase (ThermoFisher Scientific) and cloned into the pET-44 Ek/LIC Vector (Merck Millipore) by In-Fusion directional cloning (Clontech). Briefly, primers were designed to open the vector by inverse PCR (pET44_*apfA*_rev + pET44_*apfA*_for), and to amplify the genes (*apfA*_pET44_for + *apfA*_pET44_rev; *vacJ*_pET44_for + *vacJ*_pET44_rev; see Table 3). The gene specific primers did not include the start or stop codons, as these were supplied by the vector. For all primers, 15 bp extensions were added to the 5′ ends, providing reciprocal regions of complementarity (30 bp total) for In-Fusion cloning. The In-Fusion reactions were initially transformed into Stellar competent cells (Clontech) and verified before being transformed into *E. coli* BL21(DE3)pLysS competent cells, with selection on BHI agar supplemented with 100 µg/mL Ampicillin. Selected clones were screened by PCR and sequencing, and plasmids with the correct inserts were designated pApfA expr_n_ and pVacJ expr_n_ (*n* = 1–3).

For large-scale purification of expressed ApfA and VacJ proteins, the appropriate clones were inoculated into 800 mL of pre-warmed 2YT-Amp broth, using a 1/10 dilution of an ON starter culture, and incubated at 37 °C, 150 rpm, until OD_600_ reached 0.5–08. At this point, the incubation temperature was shifted to 20 °C, and 20 min later protein expression was induced by addition of 400 μL of 1 M IPTG (isopropil-β-d-1-tiogalattopiranoside). Cultures were then left to incubate ON at 20 °C, 150 rpm. Cell pellets were harvested by centrifugation (10 000 *g*, 10 min, 4 °C) and then kept on ice. Pellets were resuspended in lysis buffer (2 mL/g of pellet) and sonicated twice (80% Power, 5 pulsations, 5 min, 4 °C, 25 W effective). Cell lysates were centrifuged (40 000 *g*, 30 min, 4 °C), and supernatants were filtered on ice through 0.2 μm filters. A list of the non-commercial buffers used is provided in Additional file 2.

The recombinant proteins were isolated from the supernatants by immobilized ion metal affinity chromatography (IMAC) using the ÄKTAxpress chromatography system (GE Healthcare). Supernatants were loaded and run on HisTrap SP-HP 5 mL columns (GE Healthcare) following the manufacturer’s recommendations. The extinction coefficients of the recombinant proteins were calculated using the ExPASy ProtParam tool [45] and uploaded to the ÄKTAxpress managing program, in order to identify (by OD_280_ absorbance) the fractions containing proteins after purification. Selected protein fractions were pooled on ice, transferred to a 6–8 kD dialysis membrane (Spectra/por), and dialysed ON in 4 l PBS. Protein profiles of the purified fractions were compared to that of the initial filtered supernatant by electrophoresis under denaturing conditions using NuPAGE™ (PAGE: polyAcrylamide gel electrophoresis) 4–12% Bis–Tris Protein sodium dodecyl sulphate (SDS) Gel (ThermoFisher Scientific), run for 1 h at 180 V, and stained with Coomasie Blue.

Tags were removed from the purified proteins by cleavage with human plasma thrombin (Merck Millipore)(~2 U thrombin/μg of protein, 37 °C, ON), and separation by size exclusion chromatography (SEC). Proteins were loaded and run on Superdex 75 PG columns (GE Healthcare) using the ÄKTAxpress chromatography system. Fractions containing ApfA and VacJ proteins were identified, collected and dialyzed as described above.

### Analysis of serum samples by ELISA

Control pig serum samples were obtained from Danish pig herds participating in the specific pathogen free (SPF) system. All herd are tested for *A. pleuropneumoniae* serovars 1, 2, 5, 6, 7, 10 and 12, respectively, using ELISA (enzyme-linked immunosorbent assay) methods previously described [46]. Convalescent sera from pigs experimentally infected with *A. pleuropneumoniae* serotype 2 HK361 were also used. All animals were otherwise classified as pathogen-free. Details regarding all sera used in this study are provided in Table 4.

**Table 4:** **Animal sera used in this study**

Serum	Serovar specificity	Mode of infection
App 2	*A. pleuropneumoniae* serovar 2	Natural
App 6	*A. pleuropneumoniae* serovar 6	Natural
App 12	*A. pleuropneumoniae* serovar 12	Natural
Naive	//	Uninfected
App HK361	*A. pleuropneumoniae* HK361: serovar 2	Experimental

Antibody (IgG) responses to purified ApfA and VacJ were determined by ELISA. Briefly, 96-well flat bottom plates (TPP) were coated with 100 ng of protein/well in PBS, and incubated at 4 °C ON. Pig sera (negative controls and samples known to be positive for serovars 2, 6 and 12, respectively) were diluted threefold between 1:500 and 1:1 093 500 in diluent buffer, and incubated for 1 h at room temperature (RT). Bound antibodies were detected with peroxidase labelled rabbit anti-pig IgG (ThermoFisher Scientific) at a 1:15 000 dilution, followed by colorimetric detection (OD_450_) with TMB2 PLUS2 substrate (Kem-En-Tec Diagnostics), according to the manufacturer’s protocol. A list of the non-commercial buffers used is provided in Additional file 2. The four data sets (Control, *A. pleuropneumoniae* serotype 12, 2, 6) from ApfA and VacJ ELISA assays were transformed to log10 values and analysed by D’Agostino and Pearson ominubus normality test to verify the assumption of Gaussian distribution of the data sets (alpha = 0.05). Data sets were subsequently converted to area under curve (AUC) values and analysed by Ordinary one-way ANOVA (*P* < 0.05) to verify their statistical significance. When overall statistical significance was achieved by ANOVA, data were analysed by post hoc Holm-Sidak test to assess statistical differences between individual groups and controls. *P* < 0.05 were again considered statistically significant. All statistical analyses were performed using GraphPad Prism 6.0c (GraphPad Software, La Jolla California USA). A summary of the statistical results obtained is provided in Additional file 3.

## 2D SDS-PAGE and Western blot

Proteins from OMV isolates of *A. pleuropneumoniae* 2331 wt and Δ*nlpI* mutant, containing equal number of vesicles, were purified by Wessel-Flüge extraction [47]. Prior to IsoElectric Focusing (IEF), immobilized pH gradient (IPG) strips (pH 4–7, 7 cm, GE Life Sciences) were rehydrated in DeStreak rehydration solution (GE Life Sciences)(1.5% IPG buffer, pH 4–7, ON). The samples were applied using the cup-loading method and IEF was performed at a total of 7 kVh per strip on an Ettan IPGphor isoelectric focusing unit (GE Life Sciences). IPG strips were subsequently reduced, alkylated and proteins were separated in duplicates by SDS-PAGE as a second dimension using NuPAGE 4–12% Bis–Tris ZOOM gels (Invitrogen) in running conditions as described above for standard SDS-PAGE. The other set of gels was transferred to polyvinylidene difluoride (PVDF) membranes using an iBlot^®^ Gel Transfer Device (Thermofisher Scientific) for Western blotting. Pig sera from controls and animals that were challenged with live *A. pleuropneumoniae* HK361 cells were used as 1^a^Ab, 1:2000 dilution. A commercial Anti-Swine IgG (H + L)-Alkaline Phosphatase antibody (Sigma Aldrich) was used as 2^a^Ab, 1:10 000 dilution. Bands were revealed by 30 min incubation with dissolved SIGMAFAST™ BCIP^®^/NBT (5-bromo-4-chloro-3-indolyl-phosphate/nitro blue tetrazolium) tablets (Sigma Aldrich). A list of the non-commercial buffers used is provided in Additional file 2.

## Results

### In silico selection of conserved immunogens

A list of all potential immunogens initially identified is shown in Additional file 1. Only proteins predicted to be localised in the OM, and having ≤ 1 TMH, were included; likely secreted candidates were discarded. Details regarding predicted function and immunogenicity (based on published data) were used to narrow down the list of potential immunogens to 9 candidates (Table 2). From the 9 candidates, ApfA and VacJ proteins were selected for expression and in vitro characterization.

### Preparation of recombinant proteins and OMVs

**Figure 1:**
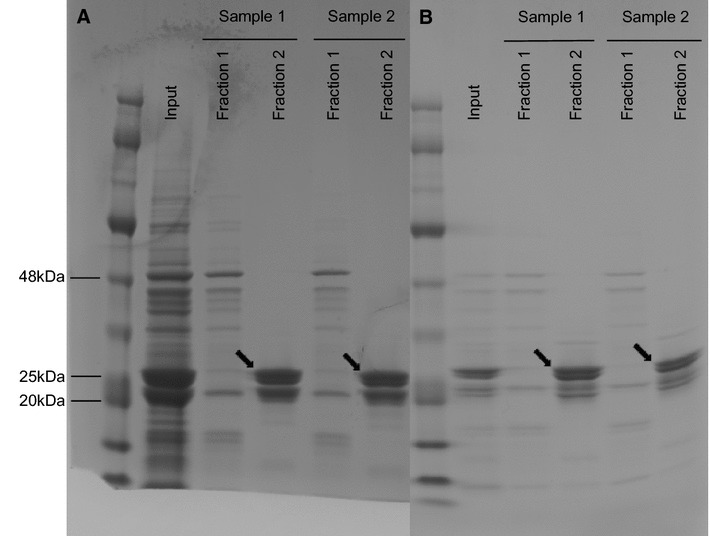
**1D SDS-PAGE analysis of purified ApfA and VacJ proteins.** Tags were removed from the expressed recombinant proteins by thrombin cleavage. Subsequently, proteins + tags were loaded on an ÄKTAxpress chromatography system and purified by size exclusion chromatography (SEC). **A** ApfA SEC; **B** VacJ SEC; Input: Proteins + cleaved tags before SEC; Fractions 1–2: Isolated fractions of purified ApfA and VacJ proteins after SEC. ApfA and VacJ proteins are indicated by black arrows. The size of selected bands of the protein marker in kilodaltons (kDa) is shown. ApfA theoretical molecular weight: 20 kDa; VacJ theoretical molecular weight: 27 kDa; Human thrombin molecular weight: 37 kDa.

Both *apfA* and *vacJ* were successfully cloned into the pET-44 Ek/LIC vector, resulting in plasmids pET-44*_apfA* and pET-44*_vacJ.* Expression of protein from *E. coli* strains harbouring these plasmids yielded a total of 46 mg and 18 mg protein for ApfA and VacJ, respectively, prior to tag removal. Following tag removal, two protein-containing fractions were isolated (Figure 1), however purified proteins were derived only from the fractions showing higher concentration and purity (i.e. the second fraction for each of ApfA and VacJ). The final protein yields were 7.4 mg for ApfA, and 4.25 mg for VacJ.

**Figure 2:**
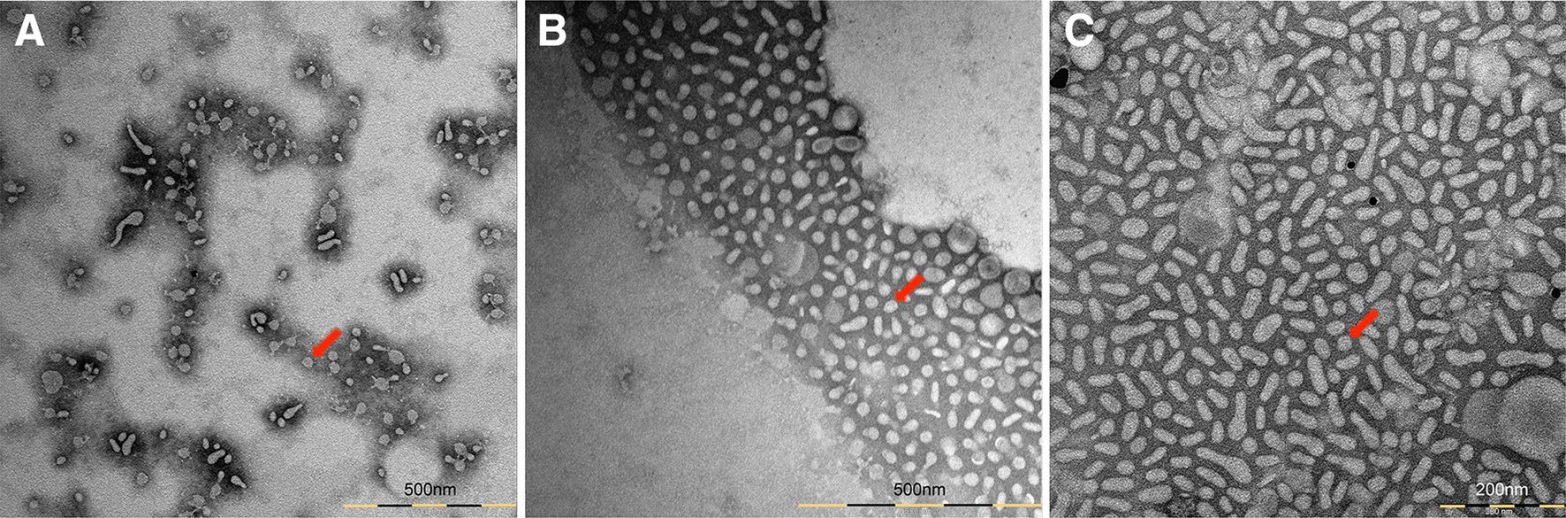
**Transmission electron microscopy (TEM) analysis of** ***A. pleuropneumoniae*** **outer membrane vesicles (OMVs).** TEM images show OMVs isolated from *A. pleuropneumoniae* MIDG2331 wild-type (**A**) and Δ*degS* (**B**), Δ*nlpI* (**C**) mutants. Red arrows indicate OMVs.

**Figure 3:**
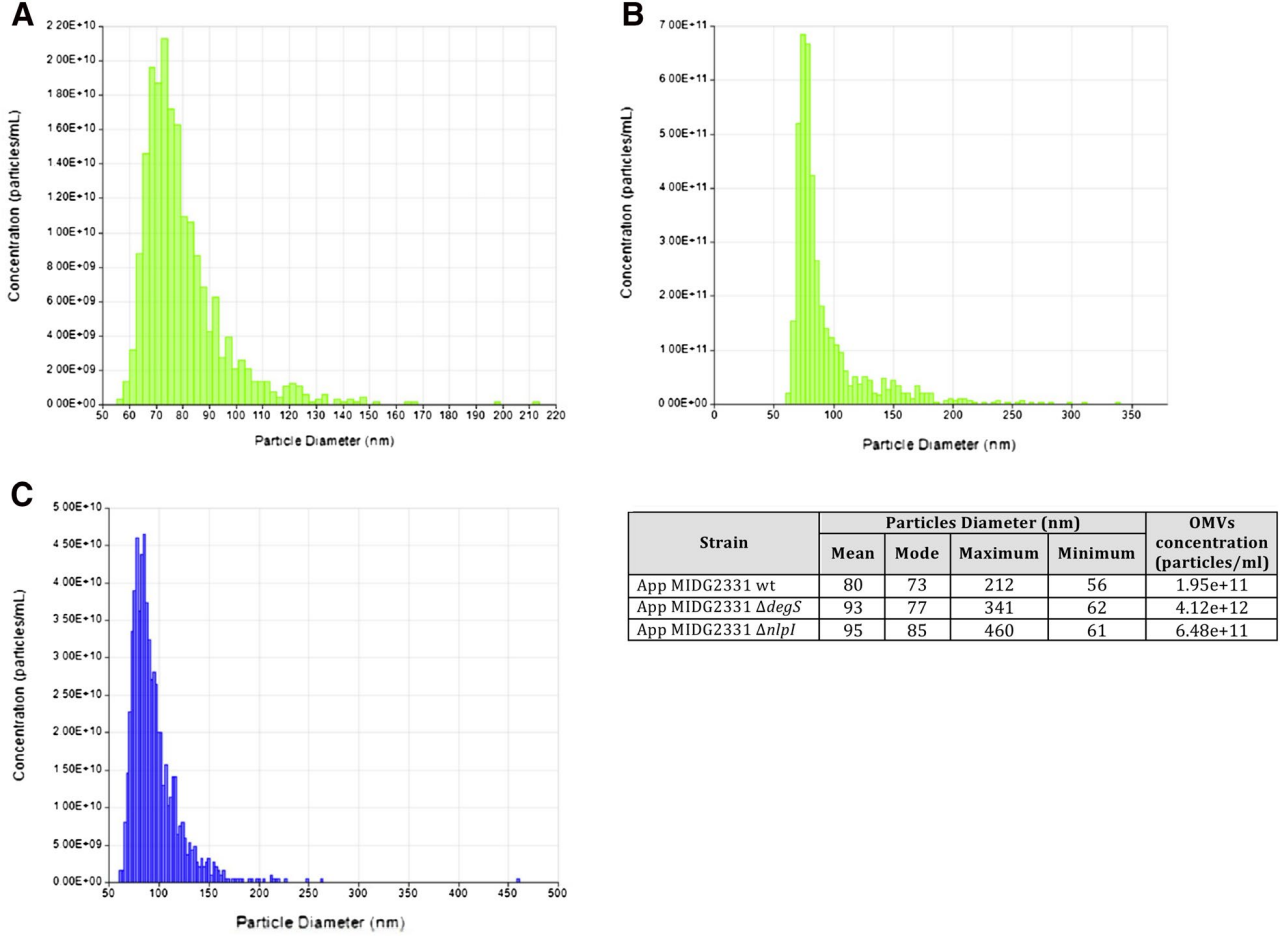
**TRPS analysis of OMVs.** TRPS analysis of OMVs from *A. pleuropneumoniae* MIDG2331 wt (**A**), Δ*degS* (**B**) and Δ*nlpI* (**C**) mutants. Size distribution and concentration of OMV samples are shown on individual graphics and resumed on the table on the right side of the figure.

The OMVs isolated by HF from wt, Δ*degS* and Δ*nlpI* mutants were analysed by TEM (Figure 2) and precisely quantified and measured by TRPS analysis (Figure 3). Both Δ*degS* and Δ*nlpI* mutants showed increased OMV secretion as compared to the wt. The size distribution of the OMVs was also affected, with a wider variation in diameter range recorded for both mutants (Figure 3).

### Assessment of in vitro immunogenicity

**Figure 4:**
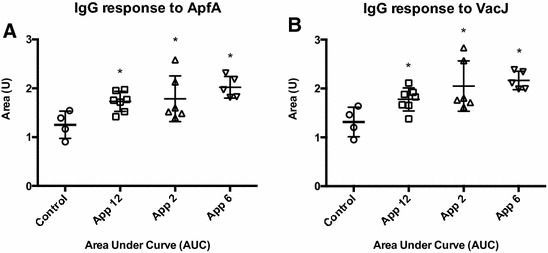
**IgG response to ApfA and VacJ proteins.** IgG response in convalescent pigs against ApfA (**A**) and VacJ (**B**) proteins. IgG response is shown as area under the curve (AUC), calculated from sera dilution curves of an ELISA assay (1:500 to 1:1 093 500 dilutions) against 100 ng/well of protein. Data sets were transformed to log_10_ and submitted to D’Agostino & Pearson omnibus normality test to verify the assumption of Gaussian distribution of the data (alpha = 0.05). After conversion of the data sets to AUC units, differences between mean AUC values of each group were analyzed by Ordinary one-way ANOVA, followed by Holm-Sidak post hoc test when overall statistical significance was determined by ANOVA (*P* < 0.05). Asterisks indicate statistical difference between individual groups and controls by Holm-Sidak (*P* < 0.05).

**Figure 5:**
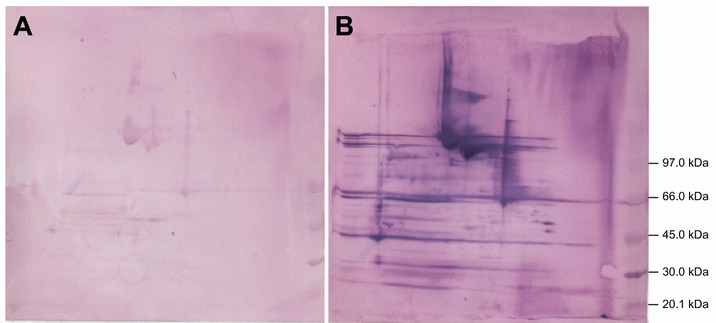
**IgG response to OMVs.** IgG response to *A. pleuropneumoniae* 2331 Δ*nlpI* OMVs was assessed by Western blot analysis using pig sera pooled from the same group of pigs, before (**A**) and after a challenge with live *A. pleuropneumoniae* HK 361 cells (**B**). Bands size in kilodaltons (kDa) of the protein marked used are provided on the right side of the figure.

Convalescent sera from animals naturally infected with *A. pleuropneumoniae* serovars 2, 6 or 12, respectively, showed a significantly higher median IgG response against both ApfA and VacJ than the control sera from uninfected pigs (Figure 4). Statistical analysis of the data sets showed the significance of the differences observed between groups in IgG levels for both ApfA and VacJ (*P* < 0.05; Additional file 3). Similarly, the Western blot (WB) analysis with sera from animals experimentally infected with *A. pleuropneumoniae* HK361 (serovar 2) showed a strong IgG response to both wt and Δ*nlpI* OMVs, and the presence of several particularly immunogenic proteins (Figure 5, only Δ*nlpI* OMVs shown).

## Discussion

The development of new vaccines is predicted to become increasingly important in the fight against bacterial pathogens, due to the emergence of multidrug-resistant strains, and the dwindling number of new antimicrobials released on the market during the recent decades [48]. Accordingly, several new strategies for the identification of potential immunogens have been proposed and implemented [16]. Most of these strategies make use of the wide range of genome sequences and bioinformatic tools, and are sometimes collectively characterized as “immunoinformatics” [49]. Despite their obvious hypothetical nature, these computational predictions and in silico analyses have proven effective in the development of at least one commercial vaccine [33].

Subcellular localization is intuitively important when selecting potential immunogens. Intracellular or periplasmic antigens are not highly accessible to the host immune system, and usually do not play a significant role in the development of adaptive immunity against extracellular bacteria [50]. On the other hand, secreted proteins are usually highly immunogenic and quite often centrally involved in the bacterial pathogenesis (e.g. toxins), but being detached from the cell they fail in many instances in providing protection against bacterial colonization when administered as vaccines [13, 15, 51]. These and other previously described considerations led us to focus our analysis on OM proteins. Some of the identified candidates are *A. pleuropneumoniae* proteins involved in iron acquisition from the host (see Table 2), and limiting access to free iron has been proven to severely affect bacterial growth during infection [52, 53]. *A. pleuropneumoniae* represents no exception, and for instance the inactivation of the *hgbA* gene has been linked to reduced virulence in vivo [54]. PepN is another interesting candidate immunogen in our list, a protease able to degrade porcine IgA and IgG [55]. It is intuitive to understand how targeting antibody degradation could be beneficial in reducing *A. pleuropneumoniae* resistance to the host immune system. However, the most promising vaccine candidate antigens that we chose to investigate in this study were ApfA and VacJ.

Type IV fimbrial subunit protein ApfA is a well characterised virulence factor in *A. pleuropneumoniae*, whose potential as an immunogen has been preliminarily investigated in swine and mouse models of infection [11, 56, 57]. ApfA is an OM pilin involved in the early stages of host colonization, and its operon has been proven to be overexpressed during cell adhesion in vitro [58]. Cell adhesion represents one of the first steps involved in the colonisation of host surfaces by a pathogenic bacterium [59], providing an ideal target for immunisation of the host with a view to blocking infection. To date, ApfA’s potential as a protective immunogen remains controversial, with some data suggesting it may be able to confer partial protection [11, 56], while others suggest ApfA may interfere with the development of an adaptive response when included in a multicomponent vaccine formulation [57]. Our data confirmed ApfA conservation among all known *A. pleuropneumoniae* serovars. Furthermore, we demonstrated that ApfA is not only expressed by *A. pleuropneumoniae* in vitro or during a challenge in an induced animal model, but also during a natural infection in pigs, eliciting a rather marked humoral immune response in animals infected by at least 3 different *A. pleuropneumoniae* serovars not previously investigated. Finally, protein domain profiling of ApfA allowed us to identify a highly conserved helical domain, common to many pilins across species, that could be potentially used as an OM “anchor” for the construction of chimeras for antigen enrichment of the bacterial OM or OMVs.

VacJ (virulence-associated chromosome locus J) is an OM lipoprotein partially conserved among several Gram-negative pathogenic bacteria, including *A. pleuropneumoniae*. VacJ is part of a proposed phospholipid transporter (VacJ/Yrb ABC transporter), the inactivation or deletion of which has recently been shown to increase OMV secretion in some species [60]. In *A. pleuropneumoniae*, VacJ has been shown to be involved in OM integrity, serum resistance and biofilm formation [61]. As its name suggests, VacJ has been proposed as potential immunogen, and proven necessary for intracellular motility of *Shigella flexneri* [62]. Furthermore, the protective potential of a VacJ immunization has been described in at least one case, for *Pasteurella multocida* [63], whose VacJ protein shares 59% homology with the *A. pleuropneumoniae* homolog. As for ApfA, our data have shown that VacJ is highly conserved, expressed by *A. pleuropneumoniae* during a natural infection, and able to induce a specific immune response in pigs.

The potential use of OMVs as immunogens has been extensively investigated in several bacterial species, including other *Pasteurellaceae* [64]. Bacterial OMVs have been shown to contain most of the antigens embedded in the OM, including LPS, toxins and other surface antigens [20]. Most notably, *A. pleuropneumoniae* OMVs were previously shown to carry Apx toxins and proteases [65]. Our data provides further insight into the immunogenicity of *A. pleuropneumoniae* OMVs, with WB profiles indicating the presence of several highly immunogenic antigens, recognised by sera from animals infected with *A. pleuropneumoniae* serovars different than that used for isolation of the OMVs. The identity of these antigenic determinants is yet to be clarified, but it is likely that they include conserved proteins and virulence factors in addition to the Apx toxins and proteases previously described [63]. Furthermore, the choice of HF as purification technique allowed the concentration of large volumes of bacterial cell-free culture supernatants, increasing OMV yield when compared to gel filtration and gradient centrifugation methods, traditionally employed in OMV isolation [27]. The ability to purify large batches of OMVs may prove very useful in the process of standardizing immunization protocols, where a large number of animals need to be vaccinated with the same sample.

In conclusion, our data support the potential of in silico identification of immunogens, and confirm the predicted high immunogenicity of *A. pleuropneumoniae* OMVs. Both ApfA and VacJ were shown to be expressed during infection by all the *A. pleuropneumoniae* serovars tested and thus may offer the long sought cross-serovar immunological potential. Accordingly, we hypothesize that OMVs, as well as ApfA and VacJ proteins, could be administered individually or as a combined vaccine for prevention of infection in pigs against all known *A. pleuropneumoniae* isolates. Nonetheless, in silico and in vitro analysis can only provide predictive information regarding the immunogenic potential of ApfA, VacJ and OMVs. In some instances in fact OM proteins have been described to produce a deleterious effect when employed as immunogens to vaccinate pigs [66], and accordingly in vivo testing in a relevant animal model (swine) will be required to verify the effective potential of ApfA, VacJ and OMVs as immunogens.

## Additional files

**Additional file 1.** **Preliminary list of** ***A. pleuropneumoniae*** **immunogen candidates.** The table lists candidate immunogens identified after the initial in silico screening of the proteomes of *A. pleuropneumoniae* JL03, L20 and AP76 strains.

**Additional file 2.** **List of non-commercial buffers used.** The table lists the formulation of all the non-commercial buffers used during this study.

**Additional file 3.** **Resume of the statistical tests performed in the study.** The table lists statistical tests and parameters used to analyse the data produced during this study.

## Abbreviations

*A. pleuropneumoniae*: *Actinobacillus pleuropneumoniae*
NAD: nicotinamide dinucleotide
OM: outer membrane
RV: reverse vaccinology
OMV: outer membrane vesicles
LPS: lipopolysaccharide
SIRS: systemic inflammatory response syndrome
TMH: trans-membrane helix
wt: wild type
BHI: brain–heart infusion
PCR: polymerase chain reaction
ON: overnight
OD: optical density
HF: hydrostatic filtration
MWCO: molecular weight cut-off
PBS: phosphate buffered saline
TEM: transmission electron microscopy
TRPS: tunable resistive pulse sensing
*E.* coli: *Escherichia coli*
IPTG: isopropil-β-d-1-tiogalattopiranoside
IMAC: ion metal affinity chromatography
PAGE: polyAcrylamide gel electrophoresis
SDS: sodium dodecyl sulphate
SPF: specific pathogen free
ELISA: enzyme-linked immunosorbent assay
RT: room temperature
AUC: area under curve
IEF: IsoElectric Focusing
IPG: immobilized pH gradient
PVDF: polyvinylidene difluoride
1^a^Ab: primary antibody
2^a^Ab: secondary antibody
BCIP/NBT: 5-bromo-4-chloro-3-indolyl-phosphate/nitro blue tetrazolium
WB: Western blot
VacJ: virulence-associated chromosome locus J
NCBI: national center for biotechnology information
CFU: colony forming unit
SEC: size exclusion chromatography

## References

[1] PROHEALTH Economic impact of *Actinobacillus pleuropneumoniae*. http://www.fp7-prohealth.eu/news-index/newsletter-november-2015/production-diseases-cost-pig-producers/. Accessed 4 Apr 2017

[2] Bossé JT, Li Y, Sárközi R, Gottschalk M, Angen Ø, Nedbalcova K, Rycroft AN, Fodor L, Langford PR (2017). A unique capsule locus in the newly designated *Actinobacillus pleuropneumoniae* serovar 16 and development of a diagnostic PCR assay. J Clin Microbiol.

[3] Bossé JT, Janson H, Sheehan BJ, Beddek AJ, Rycroft AN, Simon Kroll J, Langford PR (2002). *Actinobacillus pleuropneumoniae*: pathobiology and pathogenesis of infection. Microbes Infect.

[4] Sarkozi R, Makrai L, Fodor L (2015). Identification of a proposed new serovar of *Actinobacillus Pleuropneumoniae*: Serovar 16. Acta Vet Hung.

[5] O’Neill C, Jones SCP, Bossé JT, Watson CM, Williamson SM, Rycroft AN, Kroll JS, Hartley HM, Langford PR (2010). Prevalence of *Actinobacillus pleuropneumoniae* serovars in England and Wales. Vet Rec.

[6] Ramjeet M, Deslandes V, Gouré J, Jacques M (2008). *Actinobacillus pleuropneumoniae* vaccines: from bacterins to new insights into vaccination strategies. Anim Health Res Rev.

[7] Hensel A, Huter V, Katinger A, Raza P, Strnistschie C, Roesler U, Brand E, Lubitz W (2000). Intramuscular immunization with genetically inactivated (ghosts) *Actinobacillus pleuropneumoniae* serotype 9 protects pigs against homologous aerosol challenge and prevents carrier state. Vaccine.

[8] Negrete-Abascal E, Tenorio VR, Guerrero AL, García RM, Reyes ME, de la Garza M (1998). Purification and characterization of a protease from *Actinobacillus pleuropneumoniae* serotype 1, an antigen common to all the serotypes. Can J Vet Res.

[9] Reimer D, Frey J, Jansen R, Veit HP, Inzana TJ (1995). Molecular investigation of the role of ApxI and ApxII in the virulence of *Actinobacillus pleuropneumoniae* serotype 5. Microb Pathog.

[10] Chiers K, van Overbeke I, De Laender P, Ducatelle R, Carel S, Haesebrouck F (1998). Effects of endobronchial challenge with *Actinobacillus pleuropneumoniae* serotype 9 of pigs vaccinated with inactivated vaccines containing the Apx toxins. Vet Q.

[11] Sadilkova L, Nepereny J, Vrzal V, Sebo P, Osicka R (2012). Type IV fimbrial subunit protein ApfA contributes to protection against porcine pleuropneumonia. Vet Res.

[12] Del Pozo Sacristán R, Michiels A, Martens M, Haesebrouck F, Maes D (2014). Efficacy of vaccination against *Actinobacillus pleuropneumoniae* in two Belgian farrow-to-finish pig herds with a history of chronic pleurisy. Vet Rec.

[13] Tumamao JQ, Bowles RE, van den Bosch H, Klaasen HLBM, Fenwick BW, Storie GJ, Blackall PJ (2004). Comparison of the efficacy of a subunit and a live streptomycin-dependent porcine pleuropneumonia vaccine. Aust Vet J.

[14] Satrán P, Nedbalcová K, Kuâerová Z (2003). Comparison of protection efficacy of toxoid and whole-cell vaccines against porcine pleuropneumonia caused by endotracheal infection with *Actinobacillus pleuropneumoniae*. Acta Vet Brno.

[15] Van Overbeke I, Chiers K, Ducatelle R, Haesebrouck F (2001). Effect of endobronchial challenge with *Actinobacillus pleuropneumoniae* serotype 9 of pigs vaccinated with a vaccine containing APX toxins and transferrin-binding proteins. J Vet Med B Infect Dis Vet Public Health.

[16] Strugnell R, Zepp F, Cunningham A, Tantawichien T (2011). Vaccine antigens. Perspect Vaccinol.

[17] Seib KL, Zhao X, Rappuoli R (2012). Developing vaccines in the era of genomics: a decade of reverse vaccinology. Clin Microbiol Infect.

[18] Sette A (2010). Reverse vaccinology: developing vaccines in the era of genomics. Immunity.

[19] Rappuoli R (2000). Reverse vaccinology. Curr Opin Microbiol.

[20] Kulp A, Kuehn MJ (2010). Biological functions and biogenesis of secreted bacterial outer membrane vesicles. Annu Rev Microbiol.

[21] Kaparakis-Liaskos M, Ferrero RL (2015). Immune modulation by bacterial outer membrane vesicles. Nat Rev Immunol.

[22] Beveridge TJ, Makin SA, Kadurugamuwa JL, Li Z (1997). Interactions between biofilms and the environment. FEMS Microbiol Rev.

[23] Olsen I, Amano A (2015). Outer membrane vesicles—offensive weapons or good samaritans?. J Oral Microbiol.

[24] McBroom AJ, Johnson AP, Vemulapalli S, Kuehn MJ (2006). Outer membrane vesicle production by *Escherichia coli* is independent of membrane instability. J Bacteriol.

[25] van der Pol L, Stork M, van der Ley P (2015). Outer membrane vesicles as platform vaccine technology. Biotechnol J.

[26] Sanders H, Feavers IM (2011). Adjuvant properties of meningococcal outer membrane vesicles and the use of adjuvants in *Neisseria meningitidis* protein vaccines. Expert Rev Vaccines.

[27] Klimentova J, Stulik J (2015). Methods of isolation and purification of outer membrane vesicles from gram-negative bacteria. Microbiol Res.

[28] Nieves W, Asakrah S, Qazi O, Brown KA, Kurtz J, Aucoin DP, Mclachlan JB, Roy CJ, Morici LA (2011). A naturally derived outer-membrane vesicle vaccine protects against lethal pulmonary *Burkholderia pseudomallei* infection. Vaccine.

[29] Kim OY, Hong BS, Park K-S, Yoon YJ, Choi SJ, Lee WH, Roh T-Y, Lötvall J, Kim Y-K, Gho YS (2013). Immunization with *Escherichia coli* outer membrane vesicles protects bacteria-induced lethality via Th1 and Th17 cell responses. J Immunol.

[30] Valentine JL, Chen L, Perregaux EC, Weyant KB, Rosenthal JA, Heiss C, Azadi P, Fisher AC, Putnam D, Moe GR, Merritt JH, DeLisa MP (2016). Immunization with outer membrane vesicles displaying designer glycotopes yields class-switched, glycan-specific antibodies. Cell Chem Biol.

[31] Chen DJ, Osterrieder N, Metzger SM, Buckles E, Doody AM, DeLisa MP, Putnam D (2010). Delivery of foreign antigens by engineered outer membrane vesicle vaccines. Proc Natl Acad Sci U S A.

[32] Park K-S, Choi K-H, Kim Y-KY-S, Hong BS, Kim OY, Kim JH, Yoon CM, Koh G-Y, Kim Y-KY-S, Gho YS (2010). Outer membrane vesicles derived from *Escherichia coli* induce systemic inflammatory response syndrome. PLoS One.

[33] Vernikos G, Medini D (2014). Bexsero^®^ chronicle. Pathog Glob Health.

[34] Yu NY, Wagner JR, Laird MR, Melli G, Rey S, Lo R, Dao P, Cenk Sahinalp S, Ester M, Foster LJ, Brinkman FSL (2010). PSORTb 3.0: improved protein subcellular localization prediction with refined localization subcategories and predictive capabilities for all prokaryotes. Bioinformatics.

[35] Petersen TN, Brunak S, von Heijne G, Nielsen H (2011). SignalP 4.0: discriminating signal peptides from transmembrane regions. Nat Methods.

[36] Krogh A, Larsson B, von Heijne G, Sonnhammer EL (2001). Predicting transmembrane protein topology with a hidden Markov model: application to complete genomes. J Mol Biol.

[37] Roy A, Kucukural A, Zhang Y (2010). I-TASSER: a unified platform for automated protein structure and function prediction. Nat Protoc.

[38] Bossé JT, Li Y, Walker S, Atherton T, Fernandez Crespo R, Williamson SM, Rogers J, Chaudhuri RR, Weinert LA, Oshota O, Holden MTG, Maskell DJ, Tucker AW, Wren BW, Rycroft AN, Langford PR (2015). Identification of dfrA14 in two distinct plasmids conferring trimethoprim resistance in *Actinobacillus pleuropneumoniae*. J Antimicrob Chemother.

[39] Bossé JT, Durham AL, Rycroft AN, Kroll JS, Langford PR (2009). New plasmid tools for genetic analysis of *Actinobacillus pleuropneumoniae* and other pasteurellaceae. Appl Environ Microbiol.

[40] Bossé JT, Nash JHE, Simon Kroll J, Langford PR (2004). Harnessing natural transformation in *Actinobacillus pleuropneumoniae*: a simple method for allelic replacements. FEMS Microbiol Lett.

[41] Bossé JT, Soares-Bazzolli DM, Li Y, Wren BW, Tucker AW, Maskell DJ, Rycroft AN, Langford PR (2014). The generation of successive unmarked mutations and chromosomal insertion of heterologous genes in *Actinobacillus pleuropneumoniae* using natural transformation. PLoS One.

[42] Bossé JT, Chaudhuri RR, Li Y, Leanse LG, Fernandez Crespo R, Coupland P, Holden MTG, Bazzolli DM, Maskell DJ, Tucker AW, Wren BW, Rycroft AN, Langford PR (2016). Complete Genome sequence of MIDG2331, a genetically tractable serovar 8 clinical isolate of *Actinobacillus pleuropneumoniae*. Genome Announc.

[43] Musante L, Tataruch D, Gu D, Benito-Martin A, Calzaferri G, Aherne S, Holthofer H (2014). A simplified method to recover urinary vesicles for clinical applications, and sample banking. Sci Rep.

[44] Blundell ELCJ, Vogel R, Platt M (2016). Particle-by-particle charge analysis of DNA-modified nanoparticles using tunable resistive pulse sensing. Langmuir.

[45] Gasteiger E, Hoogland C, Gattiker A, Duvaud S, Wilkins MR, Appel RD, Bairoch A (2005) Protein identification and analysis tools on the ExPASy server. In: Proteomics Protoc. Handb. pp 571–607

[46] Nielsen R, Plambeck T, Foged NT (1991). Blocking enzyme-linked immunosorbent assay for detection of antibodies to *Actinobacillus pleuropneumoniae* serotype 2. J Clin Microbiol.

[47] Wessel D, Flügge UI (1984). A method for the quantitative recovery of protein in dilute solution in the presence of detergents and lipids. Anal Biochem.

[48] Bassetti M, Merelli M, Temperoni C, Astilean A (2013). New antibiotics for bad bugs: where are we?. Ann Clin Microbiol Antimicrob.

[49] Tomar N, De RK (2014). Immunoinformatics: a brief review. Methods Mol Biol.

[50] Ellis DW, Brodeur BR (2003) New bacterial vaccines. Springer, Berlin

[51] Spencer J, Leuzzi R, Buckley A, Irvine J, Candlish D, Scarselli M, Douce GR (2014). Vaccination against *Clostridium difficile* using toxin fragments: observations and analysis in animal models. Gut Microbes.

[52] Schaible UE, Kaufmann SH (2004). Iron and microbial infection. Nat Rev Microbiol.

[53] Parrow NL, Fleming RE, Minnick MF (2013). Sequestration and scavenging of iron in infection. Infect Immun.

[54] Shakarji L, Mikael L, Srikumar R, Kobisch M, Coulton J, Jacques M (2006). Fhua and HgbA, outer membrane proteins of *Actinobacillus pleuropneumoniae*: their role as virulence determinants. Can J Microbiol.

[55] González OG, García RM, De La Garza M, Vaca PS, Paniagua GL, Mejía R, Tenorio VR, Negrete-Abascal E (2004). *Actinobacillus pleuropneumoniae* metalloprotease: cloning and in vivo expression. FEMS Microbiol Lett.

[56] Zhou Y, Li L, Chen Z, Yuan H, Chen H, Zhou R (2013). Adhesion protein ApfA of *Actinobacillus pleuropneumoniae* is required for pathogenesis and is a potential target for vaccine development. Clin Vaccine Immunol.

[57] Shao M, Wang Y, Wang C, Guo Y, Peng Y, Liu J, Li G, Liu H, Liu S (2010). Evaluation of multicomponent recombinant vaccines against *Actinobacillus pleuropneumoniae* in mice. Acta Vet Scand.

[58] Deslandes V, Denicourt M, Girard C, Harel J, Nash JHE, Jacques M (2010). Transcriptional profiling of *Actinobacillus pleuropneumoniae* during the acute phase of a natural infection in pigs. BMC Genomics.

[59] Stones DH, Krachler AM (2016). Against the tide: the role of bacterial adhesion in host colonization. Biochem Soc Trans.

[60] Roier S, Zingl FG, Cakar F, Durakovic S, Kohl P, Eichmann TO, Klug L, Gadermaier B, Weinzerl K, Prassl R, Lass A, Daum G, Reidl J, Feldman MF, Schild S (2016). A novel mechanism for the biogenesis of outer membrane vesicles in Gram-negative bacteria. Nat Commun.

[61] Xie F, Li G, Zhang W, Zhang Y, Zhou L, Liu S, Liu S, Wang C (2016). Outer membrane lipoprotein VacJ is required for the membrane integrity, serum resistance and biofilm formation of *Actinobacillus pleuropneumoniae*. Vet Microbiol.

[62] Suzuki T, Murai T, Fukuda I, Tobe T, Yoshikawa M, Sasakawa C (1994). Identification and characterization of a chromosomal virulence gene, vacJ, required for intercellular spreading of *Shigella flexneri*. Mol Microbiol.

[63] Shivachandra SB, Kumar A, Yogisharadhya R, Viswas KN (2014). Immunogenicity of highly conserved recombinant vacj outer membrane lipoprotein of *Pasteurella multocida*. Vaccine.

[64] Pors SE, Pedersen IJ, Skjerning RB, Thøfner ICN, Persson G, Bojesen AM (2016). Outer membrane vesicles of *Gallibacterium anatis* induce protective immunity in egg-laying hens. Vet Microbiol.

[65] Negrete-Abascal E, García RM, Reyes ME, Godínez D, De La Garza M (2000). Membrane vesicles released by *Actinobacillus pleuropneumoniae* contain proteases and Apx toxins. FEMS Microbiol Lett.

[66] Van Den Bosch H, Frey J (2003). Interference of outer membrane protein PalA with protective immunity against *Actinobacillus pleuropneumoniae* infections in vaccinated pigs. Vaccine.

[67] Douidah L, De Zutter L, Baré J, De Vos P, Vandamme P, Vandenberg O, Van Den Abeele AM, Houf K (2012). Occurrence of putative virulence genes in *Arcobacter* species isolated from humans and animals. J Clin Microbiol.

[68] Kavermann H, Burns BP, Angermüller K, Odenbreit S, Fischer W, Melchers K, Haas R (2003). Identification and characterization of *Helicobacter pylori* genes essential for gastric colonization. J Exp Med J Exp Med.

[69] Plesa M, Hernalsteens JP, Vandenbussche G, Ruysschaert JM, Cornelis P (2006). The SlyB outer membrane lipoprotein of *Burkholderia multivorans* contributes to membrane integrity. Res Microbiol.

[70] Minagawa S, Ogasawara H, Kato A, Yamamoto K, Eguchi Y, Oshima T, Mori H, Ishihama A, Utsumi R (2003). Identification and molecular characterization of the Mg(2+) stimulon of *Escherichia coli*. J Bacteriol.

