# Supplementary material for: Identification and characterization of serovar-independent immunogens in Actinobacillus pleuropneumoniae
Source: Vet Res. 2017 Nov 9;48:74. doi: 10.1186/s13567-017-0479-5 (PMC5679336; doi:10.1186/s13567-017-0479-5)
Supplement: Supplementary file 1 — Additional file 1. Preliminary list of A. pleuropneumoniae immunogen candidates. The table lists candidate immunogens identified after the initial in silico screening of the proteomes of A. pleuropneumoniae JL03, L20 and AP76 strains. [file 13567_2017_479_MOESM1_ESM.docx]

**Additional file 1. Preliminary list of App immunogen candidates.**

| *Actinobacillus pleuropneumoniae* serovar 3 strain JL03 | | | | | | |
| --- | --- | --- | --- | --- | --- | --- |
| Protein ID | **Description** | | **Conserved in all serovars** | **Genetic homology among serovars (min-max)** | **Signal peptide** | **TMH** |
| amib | N-acetylmuramoyl-L-alanine amidase | | Yes | 99% | No*** | 1 |
| APJL_0265 | Probable OM protein | | Yes | 99% | Yes: CS 25-26 | 0 |
| APJL_0382 (aasP) | Serotype-specific antigen 1 precursor | | Yes | 94-99% | Yes: CS 27-28 | 0**** |
| APJL_0836 | Hypothetical protein | | Yes | 99% | No | 0 |
| APJL_0837 | Putative OM protein | | Yes | 99% | Yes: CS 22-23 | 0 |
| APJL_0839 | Membrane-bound metallopeptidase | | Yes | 99%* | Yes: CS 25_26 | 0**** |
| APJL_0979 | Large exoprotein: heme utilization or adhesion | | Yes | 99-100%* | No | 0**** |
| APJL_0980 | Hemolysin activation/secretion protein | | Yes | 99% | Yes: CS 23-24 | 0**** |
| APJL_1140 | Conserved putative lipoprotein | | Yes | 99-100% | Yes: CS 23-24 | 0 |
| APJL_1178 | Hypothetical protein | | Yes | 96-99%* | No | 0**** |
| APJL_1230 | Hypothetical protein | | Yes | 93-99% | No | 0 |
| APJL_1247 (lamB) | Maltoporin | | Yes | 82-99% | Yes: CS 23-24 | 0**** |
| APJL_1312 | Iron-regulated OM protein | | No serovar 10 | 99% | Yes: CS 22-23 | 0 |
| APJL_1729 | Hypothetical protein | | Yes | 99-100% | Yes: CS 43-44 | 0 |
| APJL_1741 | Hypothetical protein | | Yes | 95-99%* | Yes: CS 17-18 | 0 |
| APJL_1783 | OM lipoprotein A precursor | | Yes | 76-99%* | Yes: CS 21-22 | 0**** |
| APJL_1851 | Putative OM protein | | Yes | 99-100% | Yes: CS 22-23 | 0**** |
| APJL_1922 | OM receptor protein, mostly Fe transport | | Yes | 99% | No | 0 |
| APJL_1976 | Lipoprotein | | Yes | 98-100% | Yes: CS 19-20 | 0 |
| APJL_2051 | Hypothetical protein | | Yes | 99-100% | Yes: CS 22-23 | 0**** |
| fhuA | OM ferric hydroxamate receptor | | No |  | Yes: CS 23-24 | 0**** |
| frpB | Iron-regulated OM protein | | Yes | 99-100% | Yes: CS 22-23 | 0 |
| hgbA | Probable hemoglobin and hemoglobin-haptoglobin-binding protein 4 precursor | | Yes | 99% | Yes: CS 25-26 | 0 |
| hofQ | Type II secretory protein | | Yes | 99-100% | No | 0**** |
| imp | Organic solvent tolerance protein | | Yes | 99% | No | 0**** |
| irp | Iron-regulated OM protein | | Yes | 99-100% | Yes: CS 21-22 | 0 |
| lpp | OM lipoprotein | | Yes | 99-100% | Yes: CS 20-21 | 0**** |
| lppB | OM antigenic lipoprotein B | | Yes | 99% | No | 0 |
| mltA | Lytic murein transglycosylase A | | Yes | 99% | No*** | 0 |
| momP1 | Major OM protein | | Yes | 78%**,  92-99%* | Yes: CS 19-20 | 0**** |
| momP2 | Major OM protein | | Yes | 76%**, 99%* | Yes: CS 19-20 | 0 |
| ompP4 | Lipoprotein E precursor | | Yes | 99% | Yes: CS 21-22 | 0 |
| ompD | OM protein D-15 | | Yes | 99% | Yes: CS 17-18 | 0**** |
| ompP1 | OM protein P1 | | Yes | 81-99%* | Yes: CS 23-24 | 0**** |
| ompP2A2 | OM protein P2 | | Yes | 94-100%* | Yes: CS 20-21 | 0**** |
| ompP2B | OM protein P2-like protein | | Yes | 97-99%* | Yes: CS 19-20 | 0**** |
| ompW | OM protein ompw precursor | | Yes | 99-100% | Yes: CS 21-22 | 0**** |
| pal | Peptidoglycan-associated OM lipoprotein | | Yes | 97-100% | Yes: CS 18-19 | 0 |
| pgaA | Biofilm PGA synthesis protein pgaa precursor | | Yes | 99-100% | No | 0**** |
| pilF | Putative fimbrial biogenesis and twitching motility protein pilf-like protein | | Yes | 94-97% | Yes: CS 24-25 | 0 |
| plpD | Lipoprotein | | Yes | 99%* | Yes: CS 18-19 | 0**** |
| rcpA | Flp pilus assembly protein, secretin | | Yes | 99-100%* | Yes: CS 27-28 | 0**** |
| rlpB | Possible rare lipoprotein B | | Yes | 99-100% | No*** | 0 |
| smpA | Small protein A | | Yes | 98-100% | Yes: CS 20-21 | 0**** |
| tadG | Flp pilus assembly protein | | Yes | 81-89%* | No | 1 |
| tbpA1 | Transferrin-binding protein 1 precursor | | Yes | 96-99%* | Yes: CS 22-23 | 0**** |
| tbpB1 | Transferrin-binding protein 2 precursor | | No serovar 6, 12 | 78-99%* | No | 0 |
| tolC | Outer membrane protein | | Yes | 98-99% | No | 0 |
| vacJ | Vacj lipoprotein | | Yes | 98-99% | Yes: CS 25-26 | 0**** |
| *Actinobacillus pleuropneumoniae* serovar 5 strain Ap.5b.L20 | | | | | | |
| Protein ID | | **Description** | **Conserved in all serovars** | **Genetic homology among serovars**  **(min-max)** | **Signal peptide** | **TMH** |
| amiB | | Putative N-acetylmuramoyl-L-alanine amidase AmiB precursor | Yes | 99% | No*** | 1 |
| apfA | | Type 4 prepilin subunit ApfA precursor | Yes | 98-99% | No | 1 |
| APL_0246 | | Truncated transferrin-binding protein 1 precursor | Yes | 98-99% | Yes: CS 26-27 | 0 |
| APL_0257 | | Putative OM protein | Yes | 99% | Yes: CS 25-26 | 0 |
| APL_0518 | | Hypothetical protein | No | Not conserved | No | 0 |
| APL_0524 | | Predicted phage tail protein | No serovar 2,3,4,9,11,  12 | 90-96% | No | 0 |
| APL_0828 | | Hypothetical protein | Yes | 99% | No | 1 |
| APL_0829 | | Hypothetical protein | Yes | 99-100% | Yes: CS 22-23 | 0 |
| APL_0831 | | Predicted memb.-bound metallopept. | Yes | 99-100%* | No | 0**** |
| APL_0840 | | Predicted OM protein | Yes | 98-100% | No*** | 0 |
| APL_0959 | | Hemagglutinin/hemolysin-like prot. | Yes | 94-100%* | No | 0 |
| APL_1121 | | Putative lipoprotein | Yes | 99-100% | Yes: CS 23-24 | 0 |
| APL_1273 | | Putative fimbrial biogen. PilF-like prot | Yes | 95-100% | Yes: CS 25-26 | 0 |
| APL_1299 | | Predicted TonB dependent/Ligand-Gated channel | No serovar 10 | 99-100% | Yes: CS 22-23 | 0 |
| APL_1697 | | Hypothetical protein | Yes | 95-99% | No | 0 |
| APL_1748 | | OM lipoprotein A precursor | Yes | 74-99%* | Yes: CS 21-22 | 0**** |
| APL_1815 | | Hypothetical protein | Yes | 99-100% | Yes: CS 22-23 | 0**** |
| APL_1929 | | Lipoprotein | Yes | 99-100% | Yes: CS 19-20 | 0 |
| APL_1930 | | Outer membrane antigenic lipoprotein B precursor | Yes | 99-100% | Yes: CS 23-24 | 0 |
| APL_2002 | | Hypothetical protein | Yes | 99-100%* | Yes: CS 22-23 | 0 |
| APL_2016 | | Ferrioxamine B receptor precursor | No | Not conserved | No | 0 |
| cirA | | Hypothetical ABC transporter | Yes | 96-100% | No | 2 |
| cpxD | | Capsule polysaccharide export protein | Yes | 89-100% | Yes: CS 32-33 | 0 |
| D15 | | Protect. Surface antig. D15 precursor | Yes | 99-100%* | No*** | 0 |
| frpB | | Iron-regulated OM protein B | Yes | 99-100% | Yes: CS 22-23 | 0 |
| hgbA | | Hemoglobin-binding prot. A precursor | Yes | 99% | Yes: CS 25-26 | 0 |
| hofQ | | Type II secretory pathway | Yes | 99-100% | No | 0**** |
| irp | | Iron-regulated OM protein | Yes | 99%* | No | 0 |
| mltA | | Lytic murein transglycos. A precursor | Yes | 99-100% | No*** | 0 |
| ompA (ABN74505.1) | | OM protein P5 precursor | Yes | 78-99% | Yes: CS 19-20 | 0 |
| ompA (ABN74934.1) | | OM protein P5 precursor | Yes | 77-99%* | Yes: CS 19-20 | 0 |
| ompP1 | | Putative OM protein precursor | Yes | 80-99%* | Yes: CS 23-24 | 0**** |
| ompP2 | | OM protein P2 precursor | Yes | 96-100%* | Yes: CS 19-20 | 0**** |
| ompP2A | | OM protein P2 | Yes | 95-99% | Yes: CS 20-21 | 0 |
| ompP4 | | Lipoprotein E precursor | Yes | 99-100% | Yes: CS 21-22 | 0 |
| ompW | | OM protein W precursor | Yes | 99-100% | Yes: CS 21-22 | 0 |
| ostA | | Organic solvent toler. prot. precursor | Yes | 99-100% | Yes: CS 23-24 | 0 |
| palA | | OM protein precursor | Yes | 97-100% | Yes: CS 18-19 | 0 |
| pgaA | | Biofilm PGA synthesis protein PgaA precursor | Yes | 99-100% | No | 0**** |
| plpD | | lipoprotein Plp4 | Yes | 99-100%* | Yes: CS 18-19 | 0 |
| rcpA | | Rough colony protein A | Yes | 99-100%* | Yes: CS 24-25 | 0 |
| rlpB | | Putative rare lipoprotein B | Yes | 99-100% | No*** | 0 |
| slyB | | OM lipoprotein | Yes | 99-100% | Yes: CS 20-21 | 0**** |
| smpA | | Small protein A | Yes | 98-100% | Yes: 20-21 | 0**** |
| ssa1 | | Serotype-specific Antigen 1 precursor | Yes | 93-100% | Yes: CS 27-28 | 0 |
| tbpA | | Transferrin-binding protein 1 Tbp1 | Yes | 95-100%* | Yes: CS 22-23 | 0 |
| tbpB | | Transferrin-binding protein | Yes | 75-99%* | No | 0 |
| vacJ | | Lipoprotein VacJ-like protein precursor | Yes | 99-100% | Yes: CS 25-26 | 0**** |
| *Actinobacillus pleuropneumoniae* serovar 7 strain AP.7.AP76 | | | | | | |
| Protein ID | | **Description** | **Conserved in all serovars** | **Genetic homology among serovars**  **(min-max)** | **Signal peptide** | **TMH** |
| aasP | | autotransporter serine protease | Yes | 93-100% | Yes: CS 27-28 | 0**** |
| amiB | | putative N-acetylmuramoyl-L-alanine amidase AmiB precursor | Yes | 99-100% | No*** | 1 |
| APP7_0248 | | OM lipoprotein A precursor | Yes | 98-100% | Yes: CS 26-27 | 0**** |
| APP7_0259 | | OM protein P5 precursor | Yes | 99-100% | Yes: CS 25-26 | 0 |
| APP7_0502 | | phage-related minor tail protein L | No | Not conserved | No | 0 |
| APP7_0504 | | predicted phage tail protein | No | Not conserved | No | 0 |
| APP7_0885 | | hypothetical protein | Yes | 99-100% | No | 1 |
| APP7_0886 | | hypothetical protein | Yes | 99-100% | Yes: CS 22-23 | 0 |
| APP7_0888 | | predicted membrane-bound metallopeptidase | Yes | 99-100%* | Yes: CS 25-26 | 0**** |
| APP7_0897 | | predicted OM protein | Yes | 98-100% | No | 0 |
| APP7_1014 | | hemagglutinin/hemolysin-like protein | Yes | 95-100%* | No | 0**** |
| APP7_1179 | | putative lipoprotein | Yes | 99-100% | Yes: CS 23-24 | 0 |
| APP7_1217 | | hypothetical protein | Yes | 96-100%* | No*** | 0**** |
| APP7_1268 | | hypothetical protein | Yes | 93-100% | No | 1 |
| APP7_1323 | | putative fimbrial biogenesis and twitching motility protein PilF-like protein | Yes | 95-100% | Yes: CS 24-25 | 0 |
| APP7_1350 | | predicted TonB dependent/Ligand-Gated channel | No serovar 10 | 99-100% | Yes: CS 22-23 | 0 |
| APP7_1413 | | hypothetical protein | Yes | 99-100% | Yes: CS 26-27 | 1 |
| APP7_1758 | | hypothetical protein | Yes | 94-100% | Yes: CS 33-34 | 0 |
| APP7_1769 | | hypothetical protein | Yes | 97-100%* | No | 0 |
| APP7_1901 | | hypothetical protein | Yes | 99-100% | Yes: CS 22-23 | 0**** |
| APP7_1966 | | OM receptor protein | Yes | 99-100% | Yes: CS 21-22 | 0 |
| APP7_2018 | | lipoprotein | Yes | 98-100% | Yes: CS 19-20 | 0 |
| APP7_2020 | | OM antigenic lipoprotein B precursor | Yes | 99-100% | Yes: CS 23-24 | 0 |
| APP7_2042 | | Hemoglobin-haptoglobin utilization protein B precursor | Yes | 99-100% | No | 0 |
| APP7_2089 | | hypothetical protein | Yes | 99-100%* | Yes: CS 22-23 | 0**** |
| ataC | | autotransporter adhesin | No | Not conserved | No | 0 |
| cirA (0606) | | hypothetical ABC transporter ATP-binding protein | Yes | 95-100%* | No | 0 |
| cirA (0607) | | hypothetical ABC transporter ATP-binding protein | Yes | 96-100%* | No | 0 |
| d15 | | protective surface antigen D15 precursor | Yes | 99-100%* | Yes: CS 17-18 | 0**** |
| fhuA | | OM ferric hydroxamate receptor FhuA | No | Not conserved | // | 0**** |
| frpB | | iron-regulated outer membrane protein B | Yes | 99-100% | Yes: CS 22-23 | 0 |
| hecB (1015) | | Hemolysin activator protein precursor | Yes | 99-100% | No | 0 |
| hecB (1016) | | putative hemolysin activator protein | Yes | 99-100% | No*** | 0 |
| hgbA | | hemoglobin-binding protein A precursor | Yes | 99-100% | Yes: CS 25-26 | 0 |
| hofQ | | type II secretory pathway, component HofQ | Yes | 99-100% | No | 0**** |
| hpuB (2041) | | Hemoglobin-haptoglobin-binding protein A precursor | Yes | 97-100%* | No | 0 |
| hpuB (2043) | | Probable hemoglobin and hemoglobin-haptoglobin- binding protein 2 precursor | Yes | 99-100% | No | 0 |
| irp | | iron-regulated OM protein | Yes | 99-100%* | Yes: CS 21-22 | 0 |
| lamB1 | | maltoporin-1 precursor | Yes | 82-100% | Yes: CS 23-24 | 0**** |
| mltA | | membrane-bound lytic murein transglycosylase A precursor | Yes | 99-100% | No*** | 0 |
| omlA | | OM lipoprotein A precursor | Yes | 77-100%* | Yes: CS 21-22 | 0**** |
| ompA1 | | OM protein P5 precursor | Yes | (77**)93-100%* | Yes: CS 19-20 | 0 |
| ompA2 | | OM protein P5 precursor | Yes | (77**)99-100%* | Yes: CS 19-20 | 0 |
| ompP1 | | putative OM protein precursor | Yes | 78-100%* | Yes: CS 23-24 | 0**** |
| ompP2A | | OM protein P2 | Yes | 92-100% | Yes: CS 20-21 | 0**** |
| ompP4 | | lipoprotein E precursor | Yes | 99-100% | Yes: CS 21-22 | 0 |
| ompW | | OM protein W precursor | Yes | 99-100% | Yes: CS 21-22 | 0**** |
| ostA | | organic solvent tolerance protein precursor | Yes | 99-100% | Yes: CS 23-24 | 0 |
| palA | | outer membrane protein precursor PalA | Yes | 97-100% | Yes: CS 18-19 | 0 |
| pgaA | | biofilm PGA synthesis protein PgaA precursor | Yes | 99-100% | No | 0**** |
| plpD | | lipoprotein Plp4 | Yes | 99-100%* | Yes: CS 18-19 | 0**** |
| rcpA | | rough colony protein A | Yes | 99-100%* | Yes: CS 27-28 | 0**** |
| rlpB | | putative rare lipoprotein B | Yes | 99-100% | No*** | 0 |
| slyB | | outer membrane lipoprotein | Yes | 99-100% | Yes: CS 20-21 | 0**** |
| smpA | | small protein A | Yes | 98-100% | Yes: CS 20-21 | 0**** |
| tadG | | tight adherence protein G | Yes | 82-100% | No | 1 |
| tbpA | | transferrin-binding protein 1 Tbp1 | Yes | 95-100%* | Yes: CS 22-23 | 0**** |
| tbpB | | transferrin-binding protein | No serovar 6,12 | 78-100%* | No*** | 0 |
| vacj | | lipoprotein VacJ-like protein precursor | Yes | 99-100% | Yes: CS 30-31 | 0**** |

* Incomplete query coverage in at leas tone case

** The gene has 2 or more different BLAST matches and thus different percentages are presented

*** Borderline, possibly positive

**** Possibly partially transmembrane
