# Supplementary material for: Identification and characterization of serovar-independent immunogens in Actinobacillus pleuropneumoniae
Source: Vet Res. 2017 Nov 9;48:74. doi: 10.1186/s13567-017-0479-5 (PMC5679336; doi:10.1186/s13567-017-0479-5)
Supplement: Supplementary file 2 — Additional file 2. List of non-commercial buffers used. The table lists the formulation of all the non-commercial buffers used during this study. [file 13567_2017_479_MOESM2_ESM.docx]

**Additional file 2 List of non-commercial buffers used.**

| Assay | Buffer | Composition |
| --- | --- | --- |
| Protein expression  (*E. coli*) | Binding | 1x PBS, 60 mM imidazole |
|  | Elution | 1x PBS, 500 mM imidazole |
|  | Lysis | 20 mL of binding buffer + 1 tablet CMPIT without EDTA |
| Protein purification (ÄKTAxpress) | Binding | 20 mM sodium phosphate, 0.5 M NaCl, 20–40 mM imidazole, pH 7.4 |
|  | Wash | 20 mM sodium phosphate, 0.5 M NaCl, 50 mM imidazole,  pH 7.4 |
|  | Elution | 20 mM sodium phosphate, 0.5 M NaCl, 500 mM imidazole,  pH 7.4. |
| ELISA | Diluent/Blocking | 1L: 20.7 g NaCl + 5.0 g BSA + 0.5 mL Tween 20 (0.05%) + PBS (-Mg and Ca) up to 1 liter, pH 7 |
|  | Wash | 1L: 20.7 g NaCl + 1 mL Tween 20 (0.1%) + PBS (-Mg and Ca) up to 1 liter, pH 7.2 |
| SDS-PAGE | Running | 40 mL 20X NuPAGE^TM^ SDS Running Buffer (MOPS) + 760 ml ddH_2_O |
| 2D SDS-PAGE | DIGE buffer | 7 M urea, 2 M thiourea, 2% CHAPS, 2% amidosulfobetaine-14, 30 mM Tris-HCl, 1% dithiothreitol, 1% IPG buffer, 0.02% bromophenol blue, pH 4–7 |
| Western blot | PBST 0.1% | DPBS + 0.1% Tween 20 |
|  | Blocking | Blocking: 5% BSA in PBST 0.1% |
|  | Wash | Washing: PBST 0.1% |
|  | Primary Ab solution | Primary Ab solution: 15 mL Blocking buffer + Ab1 |
|  | Secondary Ab solution | Secondary Ab solution: 15 mL Blocking buffer + Ab 2 |
