# Supplementary material for: Identification and characterization of serovar-independent immunogens in Actinobacillus pleuropneumoniae
Source: Vet Res. 2017 Nov 9;48:74. doi: 10.1186/s13567-017-0479-5 (PMC5679336; doi:10.1186/s13567-017-0479-5)
Supplement: Supplementary file 3 — Additional file 3. Resume of the statistical tests performed in the study. The table lists statistical tests and parameters used to analyse the data produced during this study. [file 13567_2017_479_MOESM3_ESM.docx]

**Additional file 3 Resume of the statistical tests performed in the study**

| Data set | Statistical test | Function | Groups | *P*/alpha values |
| --- | --- | --- | --- | --- |
| ApfA ELISA  (log_10_) | D’Agostino Pearson omnibus normality test | Verify Gaussian distribution of data sets | All * | Alpha = 0.05 |
| ApfA ELISA  (AUC) | Ordinary one-way ANOVA | Verify statistical difference between mean values | All | *P* = 0.0151 |
|  | Holm-Sidak post hoc test | Assess statistical difference between individual mean values of each groups against controls | Control vs. App 12 | *P* = 0.0341 |
|  |  |  | Control vs. App 2 | *P* = 0.0341 |
|  |  |  | Control vs. App 6 | *P* = 0.0055 |
| VacJ ELISA  (log_10_) | D’Agostino Pearson omnibus normality test | Verify Gaussian distribution of data sets | All * | Alpha = 0.05 |
| VacJ ELISA  (AUC) | Ordinary one-way ANOVA | Verify statistical difference between mean values | All | *P* = 0.0072 |
|  | Holm-Sidak post hoc test | Assess statistical difference between individual mean values of each groups against controls | Control vs. App 12 | *P* = 0.0436 |
|  |  |  | Control vs. App 2 | *P* = 0.0071 |
|  |  |  | Control vs. App 6 | *P* = 0.0046 |

* Some groups of data could not be analysed due to a too low N value for the sensitivity of the test.
